# Supplementary material for: Evolutionary trajectories determine feasibility of collateral sensitivity-based antibiotic treatment strategies in critical bacterial pathogens
Source: Commun Biol. 2025 Dec 6;9:38. doi: 10.1038/s42003-025-09303-1 (PMC12783184; doi:10.1038/s42003-025-09303-1)
Supplement: Supplementary file 1 — Supplementary Information [file 42003_2025_9303_MOESM1_ESM.pdf]

Supplementary information for

Evolutionary trajectories determine feasibility of collateral sensitivity based antibiotic treatment strategies in critical bacterial pathogens

Vatsala Chauhan[1], Lisa Enkvist[1], Yuliia Chukhareva[1], Carl Damell[1], Eric Cruz Davila[1], Tasnim M. Islam[1], Greta Melander[1], Ellen Paulsson[1], Adam Sundell[1], Emily Zweifel[1], Anna af Klercker[1], Anna Knöppel[1], Gerrit Brandis[1, ✉]

[1] Department of Cell and Molecular Biology, Uppsala University 75124, Uppsala, Sweden

Corresponding author: Gerrit Brandis

✉ e-mail: gerrit.brandis@icm.uu.se

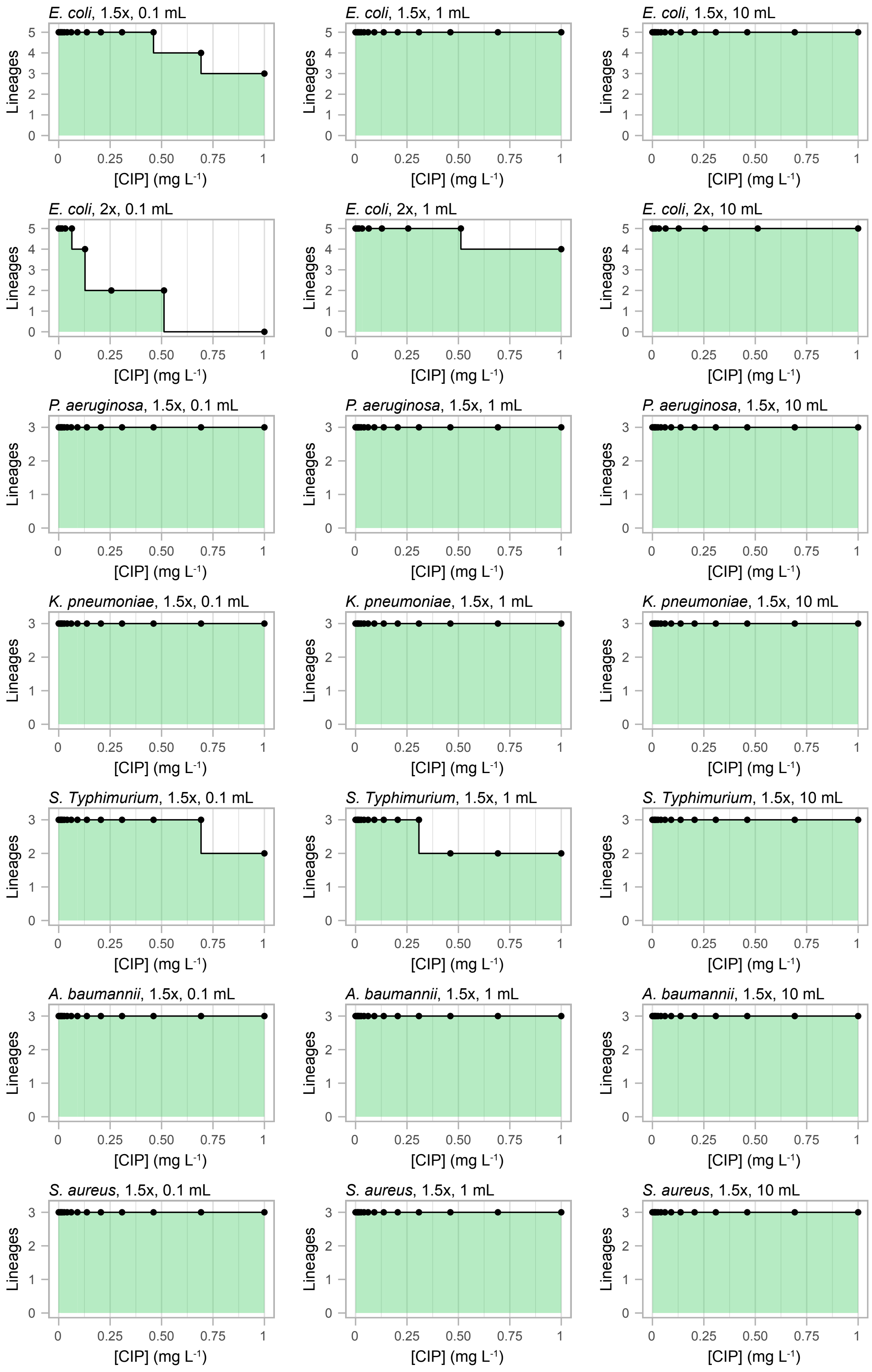

Supplementary Fig. S1: Survival of lineages during ciprofloxacin resistance evolution. Number of lineages with growth during ciprofloxacin resistance evolution. Species, stepwise increase in ciprofloxacin concentration, and culture volumes are shown above each graph.

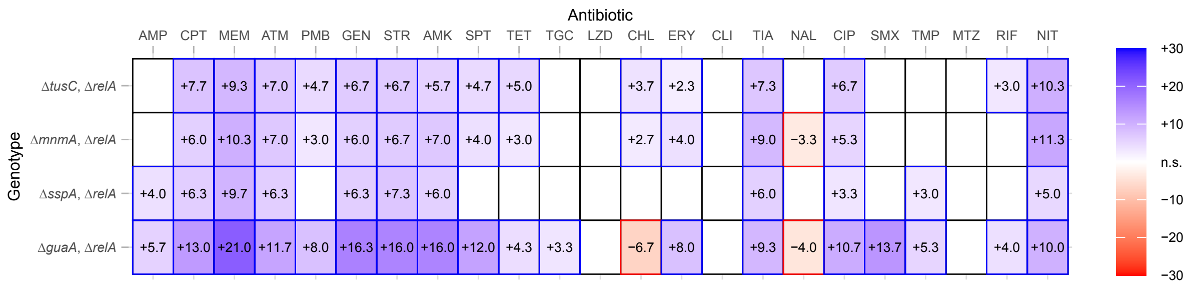

Supplementary Fig. S2: Antibiotic resistance profiles of strains with clean deletions of *tusC*, *mnmA*, *sspA*, and *guaA* in combination with deletion of *relA*. Change in the zone of inhibition between wild-type *E. coli* and constructed isolates with clean gene deletions. Increased antibiotic resistance is indicated in red, and collateral sensitivity is shown in blue. Empty fields represent no significant change. All values are averages of three biological replicates. See Supplementary Data 1 for all individual measurement values.

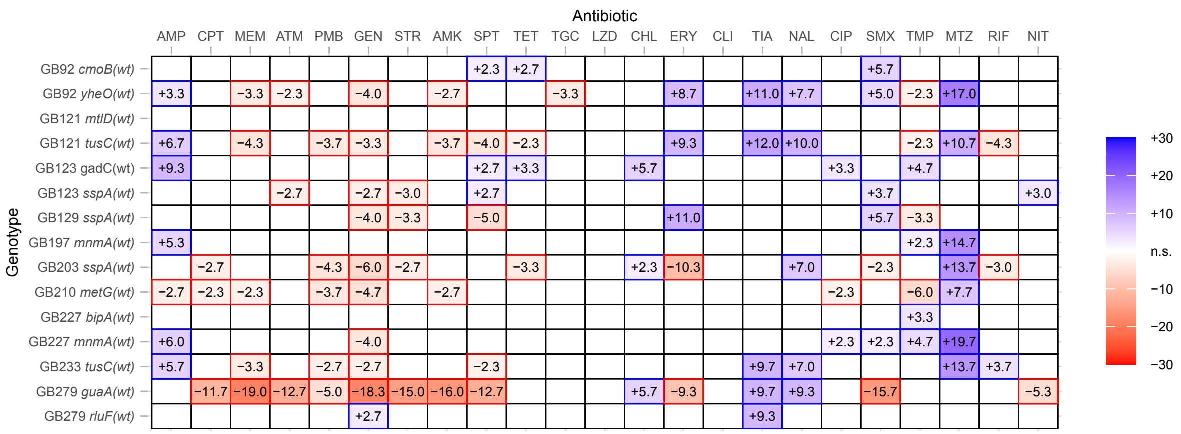

Supplementary Fig. S3: Antibiotic resistance profiles of evolved isolates with specific mutations replaced by wild-type alleles. Change in the zone of inhibition between constructed isolates and their respective parental strains. Increased antibiotic resistance is indicated in red, and collateral sensitivity is shown in blue. Empty fields represent no significant change. All values are averages of three biological replicates. See Supplementary Data 1 for all individual measurement values.

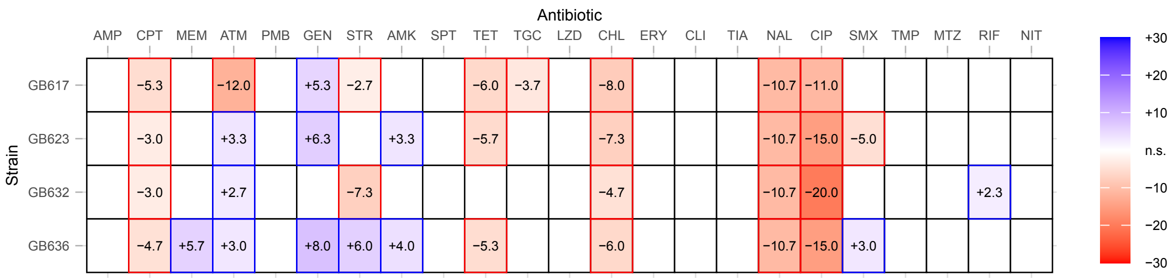

Supplementary Fig. S4: Antibiotic resistance profiles of evolved *P. aeruginosa* isolates. Change in the zone of inhibition between wild-type *P. aeruginosa* and evolved isolates. Increased antibiotic resistance is indicated in red, and collateral sensitivity is shown in blue. Empty fields represent no significant change. All values are averages of three biological replicates. See Supplementary Data 1 for all individual measurement values.

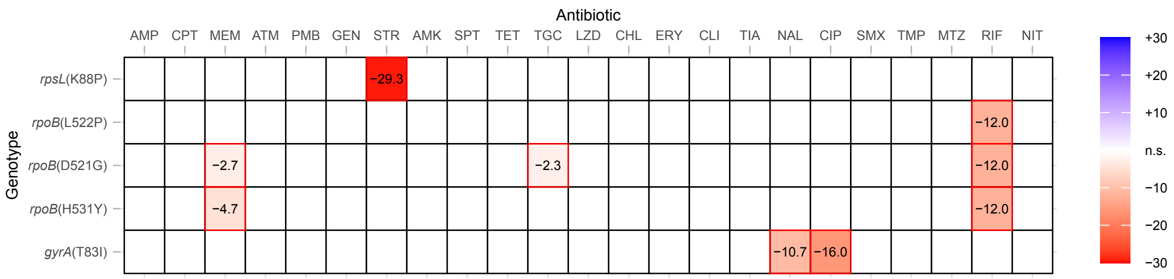

Supplementary Fig. S5: Antibiotic resistance profiles of selected *P. aeruginosa* isolates. Change in the zone of inhibition between wild-type *P. aeruginosa* and isolates with point mutations in antibiotic target genes. Increased antibiotic resistance is indicated in red, and collateral sensitivity is shown in blue. Empty fields represent no significant change. All values are averages of three biological replicates. See Supplementary Data 1 for all individual measurement values.

Supplementary Table S1. Overview over evolution experiment

Volume (mL)	CIP increase	Lineage	Endpoint CIP (mg L^{-1})	Isolate	GEN (mm)[a]	CIP MIC (mg L^{-1})[b]	WGS
0.1	1.5x	1	1.038	1	24.3	-	-
0.1	1.5x	1	1.038	2	24.3	1	GB88
0.1	1.5x	1	1.038	3	24.7		-
0.1	1.5x	1	1.038	4	24.3		-
0.1	1.5x	1	1.038	5	24.7		-
0.1	1.5x	2	0.461	1	25.3		-
0.1	1.5x	2	0.461	2	25.7	0.512	GB91
0.1	1.5x	2	0.461	3	29.7	0.512	GB92
0.1	1.5x	2	0.461	4	27.7		-
0.1	1.5x	2	0.461	5	27.7		-
0.1	1.5x	3	0.692	1	24.7		-
0.1	1.5x	3	0.692	2	25		-
0.1	1.5x	3	0.692	3	25.3	1	GB95
0.1	1.5x	3	0.692	4	25		-
0.1	1.5x	3	0.692	5	24.7		-
0.1	1.5x	4	1.038	1	27.7		-
0.1	1.5x	4	1.038	2	27.3		-
0.1	1.5x	4	1.038	3	27.3		-
0.1	1.5x	4	1.038	4	27.3	1	GB197
0.1	1.5x	4	1.038	5	27		-
0.1	1.5x	5	1.038	1	29.3		-
0.1	1.5x	5	1.038	2	29		-
0.1	1.5x	5	1.038	3	29.7		-
0.1	1.5x	5	1.038	4	29.7		-
0.1	1.5x	5	1.038	5	30		-
0.1	2x	1	0.512	1	23.3		-
0.1	2x	1	0.512	2	24.3		-
0.1	2x	1	0.512	3	25	0.256	GB98
0.1	2x	1	0.512	4	42	1	GB279
0.1	2x	1	0.512	5	25		-
0.1	2x	2	0.128	1	25.7	0.128	GB99
0.1	2x	2	0.128	2	25.3		-
0.1	2x	2	0.128	3	25.7		-
0.1	2x	2	0.128	4	35	0.256	GB281
0.1	2x	2	0.128	5	34.7		-
0.1	2x	3	0.512	1	25.3		-
0.1	2x	3	0.512	2	25.7		-
0.1	2x	3	0.512	3	25.3	0.256	GB104
0.1	2x	3	0.512	4	25		-
0.1	2x	3	0.512	5	25.7		-
0.1	2x	4	0.128	1	25.7	0.128	GB204
0.1	2x	4	0.128	2	25.7		-
0.1	2x	4	0.128	3	25.3		-
0.1	2x	4	0.128	4	25.7		-
0.1	2x	4	0.128	5	25.3		-

Supplementary Table S1. Continued

Volume (mL)	CIP increase	Lineage	Endpoint CIP (mg L^{-1})	Isolate	GEN (mm)[a]	CIP MIC (mg L^{-1})[b]	WGS
0.1	2x	5	0.064	1	24.7	0.064	GB209
0.1	2x	5	0.064	2	29.3	0.064	GB210
0.1	2x	5	0.064	3	25.3	-	-
0.1	2x	5	0.064	4	24.7	-	-
0.1	2x	5	0.064	5	24.3	-	-
1	1.5x	1	1.038	1	24.3	2	GB105
1	1.5x	1	1.038	2	25.7	-	-
1	1.5x	1	1.038	3	24.3	-	-
1	1.5x	1	1.038	4	25.3	-	-
1	1.5x	1	1.038	5	25.7	-	-
1	1.5x	2	1.038	1	25	2	GB108
1	1.5x	2	1.038	2	24.3	-	-
1	1.5x	2	1.038	3	23.3	-	-
1	1.5x	2	1.038	4	25	-	-
1	1.5x	2	1.038	5	25	-	-
1	1.5x	3	1.038	1	24.7	2	GB111
1	1.5x	3	1.038	2	24.3	-	-
1	1.5x	3	1.038	3	24.7	-	-
1	1.5x	3	1.038	4	24.3	-	-
1	1.5x	3	1.038	5	24.7	-	-
1	1.5x	4	1.038	1	24.3	-	-
1	1.5x	4	1.038	2	23.7	-	-
1	1.5x	4	1.038	3	24.7	1	GB216
1	1.5x	4	1.038	4	24	-	-
1	1.5x	4	1.038	5	24.7	-	-
1	1.5x	5	1.038	1	25.3	1	GB219
1	1.5x	5	1.038	2	25.3	-	-
1	1.5x	5	1.038	3	25.3	-	-
1	1.5x	5	1.038	4	25.7	-	-
1	1.5x	5	1.038	5	25.3	-	-
1	2x	1	1.024	1	24.3	-	-
1	2x	1	1.024	2	24.7	-	-
1	2x	1	1.024	3	24.7	2	GB116
1	2x	1	1.024	4	24	-	-
1	2x	1	1.024	5	24.3	-	-
1	2x	2	1.024	1	24.7	-	-
1	2x	2	1.024	2	25.3	2	GB118
1	2x	2	1.024	3	25.7	-	-
1	2x	2	1.024	4	24.3	-	-
1	2x	2	1.024	5	25	-	-
1	2x	3	0.512	1	28.7	-	-
1	2x	3	0.512	2	29.3	0.512	GB121
1	2x	3	0.512	3	23.7	0.512	GB122
1	2x	3	0.512	4	29.3	-	-
1	2x	3	0.512	5	24.3	-	-

Supplementary Table S1. Continued

Volume (mL)	CIP increase	Lineage	Endpoint CIP (mg L^{-1})	Isolate	GEN (mm)[a]	CIP MIC (mg L^{-1})[b]	WGS
1	2x	4	1.024	1	28.7	-	-
1	2x	4	1.024	2	28.3	-	-
1	2x	4	1.024	3	27.3	-	-
1	2x	4	1.024	4	29.3	0.512	GB227
1	2x	4	1.024	5	27.7	-	-
1	2x	5	1.024	1	28	-	-
1	2x	5	1.024	2	27.7	-	-
1	2x	5	1.024	3	28	-	-
1	2x	5	1.024	4	27.3	-	-
1	2x	5	1.024	5	28.3	0.512	GB233
10	1.5x	1	1.038	1	27.3	1	GB123
10	1.5x	1	1.038	2	27.7	-	-
10	1.5x	1	1.038	3	26.7	-	-
10	1.5x	1	1.038	4	26.3	-	-
10	1.5x	1	1.038	5	27	-	-
10	1.5x	2	1.038	1	25.3	-	-
10	1.5x	2	1.038	2	24.3	-	-
10	1.5x	2	1.038	3	24.7	2	GB128
10	1.5x	2	1.038	4	25.3	-	-
10	1.5x	2	1.038	5	25.3	-	-
10	1.5x	3	1.038	1	28.7	2	GB129
10	1.5x	3	1.038	2	28.3	-	-
10	1.5x	3	1.038	3	27.7	-	-
10	1.5x	3	1.038	4	27.3	-	-
10	1.5x	3	1.038	5	27.7	-	-
10	1.5x	4	1.038	1	24.7	-	-
10	1.5x	4	1.038	2	25	2	GB235
10	1.5x	4	1.038	3	24.7	-	-
10	1.5x	4	1.038	4	25	-	-
10	1.5x	4	1.038	5	24.3	-	-
10	1.5x	5	1.038	1	25.3	2	GB239
10	1.5x	5	1.038	2	24.7	-	-
10	1.5x	5	1.038	3	24.7	-	-
10	1.5x	5	1.038	4	24.3	-	-
10	1.5x	5	1.038	5	24.3	-	-
10	2x	1	1.024	1	24.3	-	-
10	2x	1	1.024	2	24.7	-	-
10	2x	1	1.024	3	24.7	2	GB134
10	2x	1	1.024	4	24.3	-	-
10	2x	1	1.024	5	24.3	-	-
10	2x	2	1.024	1	24.3	1	GB135
10	2x	2	1.024	2	24.3	-	-
10	2x	2	1.024	3	24.7	-	-
10	2x	2	1.024	4	24	-	-
10	2x	2	1.024	5	23.7	-	-

Supplementary Table S1. Continued

Volume (mL)	CIP increase	Lineage	Endpoint CIP (mg L^{-1})	Isolate	GEN (mm)[a]	CIP MIC (mg L^{-1})[b]	WGS
10	2x	3	1.024	1	24.7	-	-
10	2x	3	1.024	2	25.3	-	-
10	2x	3	1.024	3	25.7	1	GB140
10	2x	3	1.024	4	25.7	-	-
10	2x	3	1.024	5	25.3	-	-
10	2x	4	1.024	1	24.7	-	-
10	2x	4	1.024	2	25	-	-
10	2x	4	1.024	3	25.3	1	GB246
10	2x	4	1.024	4	25.3	-	-
10	2x	4	1.024	5	25.3	-	-
10	2x	5	1.024	1	24.3	-	-
10	2x	5	1.024	2	25	-	-
10	2x	5	1.024	3	24.7	-	-
10	2x	5	1.024	4	25.3	1	GB252
10	2x	5	1.024	5	25.7	-	-

[a] Average diameter of three independent gentamicin disc diffusion test. Wild-type *E. coli*: 24 ± 1 mm. See Supplementary Data 1 for individual measurements.

[b] Ciprofloxacin MIC based on broth microdilution assay in triplicates. Wild-type *E. coli*: 0.016 mg L^{-1}. See Supplementary Data 1 for individual measurements.

Supplementary Table S2. Full genotypes of evolved *E. coli* isolates

**Strain**	**Genotype based on whole genome sequencing analysis**
GB88	*gyrA*(Asp87Tyr), *marR*(ΔGlu136-Leu139), *pdeR*(Ala283Gly), Δe14
GB91	*gyrA*(ΔSer83), *soxR*(Leu148fs), DUP*insH12-insH6*(72kb)
GB92	*gyrA*(ΔSer83), *marR*(ΔTyr137-Lys140), c*moB*(Glu51*), *yheO*(A168<>*insH*)
GB95	*gyrA*(Asp82Gly + Ser83Asp), *marR*(Val84Glu), *yeeN*(Asp40Asp)
GB98	*gyrA*(Asp87Tyr), *serA*(Thr268fs), *maa*(G-142T)
GB99	*gyrA*(Val48Glu), *tufA*(Ala187fs)
GB104	*gyrA*(ΔSer83), *soxR(*Asp137fs)
GB105	*gyrA*(Asp87Gly), *soxR*(ΔArg127), *rpoB*(ΔLeu989-Gln1038)
GB108	*gyrA*(Asp87Gly), *soxR*(ΔArg127), *rpoB*(Gln1268Leu)
GB111	*gyrA*(Ser83Leu), *parC*(Ser80Ile)
GB116	*gyrA*(Asp87Tyr), *soxR*(Asp137fs), Δ*thrV*
GB118	*gyrA*(Ser83Glu), *soxR*(Asp137Ala), Δ*thrV*, *nfsA*(Leu97Leu)
GB121	*gyrA*(ΔSer83), *marR*(Leu17fs), *tusC*(ΔGlu102), *mtlD*(Leu138fs)
GB122	*gyrA*(ΔSer83), *iscS*(Ser38fs), *ybcL*(T-107C)
GB123	*gyrA*(Ala119Glu), *acrR*(Gln14*), *marR*(Val84Gly), *gadC*(Ala113Thr), *sspA*(Gln57fs)
GB128	*gyrA*(Ser83Leu), *marR*(Glu136fs), *fimA*(INV(A-424-A-128 + G-340<>*insH*))
GB129	*gyrA*(Ser83Leu), *parC*(Ala117Glu), *sspA*(Ile173fs)
GB134	*gyrA*(Ser83Leu), *marR*(ΔT-37-A-23)
GB135	*gyrA*(Asp87Tyr), *acrR*(ΔVal19-Arg22), *marR*(Leu75Arg)
GB140	*gyrA*(Gly81Cys), *soxR*(Asn134fs), *ynfD*(C-39<>*insCD)*
GB197	*gyrA*(ΔSer83), *acrR*(Leu34Gln), *marR*(Leu87fs), *mnmA*(Ile106Ser), *fimA*(INV(A-424-A-128))
GB203	*gyrA*(ΔSer83), *acrR*(Trp50Arg), *marR*(Arg77Leu), *sspA*(C-356A), *fimA*(INV(A-424-A-128))
GB204	*gyrA*(ΔSer83)
GB209	*marR*(ΔLeu29-Glu31), *nimR*(Phe88Val), Δ*valT-lysW-valZ-lysYZQ*, *fimA*(INV(A-424-A-128))
GB210	*marR*(ΔLeu29-Glu31), *nimR*(Phe88Val), *metG*(Phe305Cys), *fimA*(INV(A-424-A-128))
GB216	*gyrA*(Asp87Gly), *acrR*(Lys53fs), s*oxR*(ΔA-24), *mdtE*(ΔVal185), *dgcE*(Gly803Asp)
GB219	*gyrA*(Gly81Cys), *acrR*(Thr5Ala), *marR(*Arg86fs), *gltP*(A-115T), Δ*glnV*
GB227	*gyrA*(ΔSer83), *acrR*(G220<>insH), *mnmA*(Gln59*), *bipA*(Leu168fs), *fimA*(INV(A-424-A-128))
GB233	*gyrA*(ΔSer83), *acrR*(DUPAla20), *marR*(Glu136<>PheGlu), *tusC*(Gly21Val), *fimA*(INV(A-424-A-128))
GB235	*gyrA*(Ser83Leu + Asp87Gly), *soxR*(Gly121Ser), *fimA*(ΔT-390)
GB239	*gyrA*(Ser83Leu), *parC(*Ser80Arg), *ygdT*(Asp8<>*insAB*), *fimA*(INV(A-424-A-128 + C-412T))
GB246	*gyrA*(Ser83Leu), *soxR*(Arg138fs), *trkH*(Gly157Arg), *fimA*(INV(A-424-A-128))
GB252	*gyrA*(Ala119Glu), *gyrB*(Glu466Asp), *parE*(DUPSer458)
GB279	*gyrA*(ΔSer83), *soxR*(Arg20Leu), *guaA*(Asp462Tyr), *rluF*(Cys19Phe)
GB281	*gyrA*(Val48Glu), *tufA*(Ala187fs), DUP*insH1-insH3*(414kb)

Supplementary Table S3. Analysis of clinically relevant resistance mutations in evolved isolates.

Gene	GEN Phenotype	Mutated	Wild-type	P-value[a]
gyrA	CS	10	1	0.5361
	Non-CS	23	1	
parC	CS	1	10	1.00
	Non-CS	2	22	
acrR	CS	5	6	0.0767
	Non-CS	3	21	
marR	CS	6	5	0.1297
	Non-CS	6	18	
soxR	CS	1	10	0.1140
	Non-CS	10	14	

[a] P-value based on a two-sided Fisher's exact test.

Supplementary Table S4. Ciprofloxacin-resistance analysis of *E. coli* genomes from the BV-BRC database.

**Class**	**Mutations**	***gyrA***		***parC***		**Isolates**
		Ser83	Asp87	Ser80	Glu84	
CIP^S	0	-	-	-	-	403
CIP^R	1	Ser83Ala	-	-	-	5
CIP^R	1	Ser83Leu	-	-	-	46
CIP^R	1	-	Asp87Asn	-	-	1
CIP^R	1	-	Asp87Gly	-	-	1
CIP^R	1	-	Asp87Tyr	-	-	3
CIP^R	2	Ser83Leu	-	Ser80Arg	-	2
CIP^R	2	Ser83Leu	-	Ser80Ile	-	5
CIP^R	2	-	Asp87Tyr	Ser80Arg	-	2
CIP^R	3	Ser83Leu	Asp87Asn	Ser80Arg	-	1
CIP^R	3	Ser83Leu	Asp87Asn	Ser80Ile	-	259
CIP^R	3	Ser83Leu	Asp87Gly	Ser80Ile	-	1
CIP^R	3	Ser83Leu	Asp87His	Ser80Ile	-	1
CIP^R	3	Ser83Leu	Asp87Tyr	Ser80Ile	-	10
CIP^R	3	Ser83Leu	-	Ser80Ile	Glu84Val	1
CIP^R	4	Ser83Leu	Asp87Asn	Ser80Ile	Glu84Ala	2
CIP^R	4	Ser83Leu	Asp87Asn	Ser80Ile	Glu84Gly	19
CIP^R	4	Ser83Leu	Asp87Asn	Ser80Ile	Glu84Val	71
CIP^R	4	Ser83Leu	Asp87Tyr	Ser80Ile	Glu84Gly	1
CIP^R	4	Ser83Leu	Asp87Tyr	Ser80Ile	Glu84Lys	1

Supplementary Table S5. Mutational analysis of *E. coli* genomes from the BV-BRC database.

**Gene**	**Class**	**Mutated**	**Wild-type**	**P-value**[a]
guaA	CIP^R	14	418	0.8512
	CIP^S	15	388	
metG	CIP^R	29	403	0.6878
	CIP^S	30	373	
mnmA	CIP^R	15	417	0.0747
	CIP^S	25	378	
sspA	CIP^R	2	430	0.0321
	CIP^S	9	394	
tusC	CIP^R	6	426	0.7832
	CIP^S	7	396	

[a] P-value based on a two-sided Fisher's exact test.

Supplementary Table S6. Exponential growth rates of evolved *P. aeruginosa* isolates.

**Strain**	**Type**	**Doubling time ± SD (min)**	**P-value[a]**
GB478	Wild-type	21.5 ± 0.6	-
GB613	CS	28.1 ± 2.0	0.006
GB617	CS	19.5 ± 0.8	0.025
GB619	CS	27.0 ± 1.5	0.004
GB623	CS	24.6 ± 0.8	0.006
GB626	CS	19.4 ± 0.6	0.015
GB630	CS	21.1 ± 0.3	0.398
GB632	Non-CS	20.4 ± 0.5	0.061
GB636	CS	31.1 ± 1.8	0.001
GB638	CS	33.0 ± 1.9	0.001

[a] P-value based on a two-sided t-test.

Supplementary Table S7. Full genotypes of evolved *P. aeruginosa* isolates

**Strain**	**Genotype based on whole genome sequencing analysis**
GB617	*gyrB*(Pro749Ser), *mexR*(Arg223fs)
GB623	Δ1768 bp (*nfxB* T-96 – *morA* C865)
GB632	*gyrA*(Thr83Ile), *amrR*(ΔAsp83-Leu85), *pilR*(Thr422fs)
GB636	*nfxB*(ΔLeu23-Leu29), *morA*(Ser663Arg), *pilR*(ΔPhe348-Val352)

Supplementary Table S8. Antibiotic discs.

**Antibiotic**	**Quantity (µg)**
Ampicillin	10
Ceftaroline	30
Meropenem	10
Aztreonam	30
Polymyxin B	300
Gentamicin	30
Streptomycin	300
Amikacin	30
Spectinomycin	25
Tetracyclin	30
Tigecyclin	15
Linezolid	30
Chloramphenicol	30
Erythromycin	30
Clindamycin	10
Tiamulin	30
Nalidixic Acid	30
Ciprofloxacin	5
Sulfamethoxazole	100
Trimethoprim	5
Metronidazole	50
Rifampicin	30
Nitrofurantoin	300

Supplementary Table S9. Ciprofloxacin concentrations during evolution experiments.

**Cycle**	**Ciprofloxacin concentration (mg L^{-1})**	
	**1.5x increase**	**2x increase**
1	0	0
2	0.008	0.008
3	0.012	0.016
4	0.018	0.032
5	0.027	0.064
6	0.041	0.128
7	0.061	0.256
8	0.091	0.512
9	0.137	1.024
10	0.205	
11	0.308	
12	0.461	
13	0.692	
14	1.038	
