# Supplementary material for: Evolutionary trajectories determine feasibility of collateral sensitivity-based antibiotic treatment strategies in critical bacterial pathogens
Source: Commun Biol. 2025 Dec 6;9:38. doi: 10.1038/s42003-025-09303-1 (PMC12783184; doi:10.1038/s42003-025-09303-1)
Supplement: Supplementary file 2 — Description of Additional Supplementary Materials [file 42003_2025_9303_MOESM2_ESM.pdf]

Description of Additional Supplementary Files

File name: Supplementary Data 1

Description: Collection of all individual measurements.

File name: Supplementary Data 2

Description: Raw data for growth curves.

File name: Supplementary Data 3

Description: The source data behind the graphs in figure 1.

File name: Supplementary Data 4

Description: The source data behind the graphs in figure 2.

File name: Supplementary Data 5

Description: The source data behind the graphs in figure 3.

File name: Supplementary Data 6

Description: The source data behind the graphs in figure 4.

File name: Supplementary Data 7

Description: The source data behind the graphs in figure 5.

File name: Supplementary Data 8

Description: The source data behind the graphs in figure 6.
